# Next-Generation Sequencing in Translational Sarcoma Research: Biological Insights and Emerging Directions

**DOI:** 10.1007/s11912-026-01828-w

**Published:** 2026-09-17

**Authors:** Alessandro De Vita, Sara Violani, Giacomo Miserocchi, Elena Xerxa, Jürgen Bajorath, Silvia Vanni

**Affiliations:** 1 Preclinic and Osteoncology Unit, Biosciences Laboratory, IRCCS Istituto Romagnolo per lo Studio dei Tumori (IRST) “Dino Amadori”, Via Piero Maroncelli 40, Meldola, 47014 Italy; 2 Department of Life Science Informatics and Data Science, B-IT (Bonn-Aachen International Center for Information Technology), Bonn, Germany; 3 Lamarr Institute for Machine Learning and Artificial Intelligence, University of Bonn, Friedrich- Hirzebruch-Allee 5/6, Bonn, D-53115 Germany

**Keywords:** Next-generation sequencing, Sarcoma, Mutation discovery, Fusion genes, Biomarkers, Tumor heterogeneity, Precision oncology

## Abstract

**Purpose of Review:**

Sarcomas are rare and heterogeneous malignancies for which molecular diagnostics increasingly complement histopathology, immunohistochemistry, FISH, and RT-PCR. This review summarizes the main research applications of next-generation sequencing (NGS) in sarcoma, with particular attention to mutation and variant discovery, fusion detection, biomarker development, assay validation, multi-omics integration, and the boundaries between exploratory research and clinical implementation.

**Recent Findings:**

NGS-based approaches have expanded the detection of recurrent and rare alterations across sarcoma subtypes, including subtype-defining fusions, copy-number changes, actionable kinase rearrangements, and alterations in tumor suppressor and cell-cycle pathways. However, the clinical utility of NGS remains context-dependent and should be interpreted according to tumor subtype, sample quality, available orthogonal methods, multidisciplinary expertise, and current precision-oncology recommendations.

**Summary:**

NGS is a powerful tool in translational sarcoma research and, in selected clinical scenarios, supports diagnosis, molecular classification, and therapeutic decision-making. Its use should be presented with appropriate caution, acknowledging technical limitations, the need for expert reference-center interpretation, and the complementary role of established diagnostic techniques.

**Supplementary Information:**

The online version contains supplementary material available at https://doi.org/10.1007/s11912-026-01828-w.

## Introduction

Sarcomas are a diverse group of malignant tumors arising from mesenchymal tissues, encompassing over 70 histological subtypes that affect bone, cartilage, muscle, and connective tissue [23, 30, 72]. Their rarity and heterogeneity have historically limited research progress, making them some of the most challenging malignancies to study and treat.

Traditional diagnostic modalities, such as fluorescence in situ hybridization (FISH) and reverse-transcription polymerase chain reaction (RT-PCR), have contributed substantially to the identification of specific chromosomal translocations and fusion genes in sarcomas, including *EWSR1*-*FLI1*/*EWSR1*-*ERG* in Ewing sarcoma, *SS18*-*SSX1*/2/4 in synovial sarcoma, *FUS*-*DDIT3* in myxoid liposarcoma, *COL1A1*-*PDGFB* in dermatofibrosarcoma protuberans, and *NAB2*-*STAT6* in solitary fibrous tumor, PAX3-FOXO1 in alveolar rhabdomyosarcoma [18, 60, 69]. Nevertheless, these techniques are generally most informative when a specific diagnostic hypothesis is already present and when the expected gene partner or breakpoint can be targeted; they may be less efficient for rare, cryptic, or novel rearrangements and for broad molecular characterization [45, 60].

The introduction of next-generation sequencing (NGS) technologies has revolutionized the study of sarcomas by enabling parallel, high-throughput sequencing of DNA and RNA, offering an unbiased view of the genomic landscape [17, 56]. Through whole-genome, whole-exome, and targeted sequencing, NGS has uncovered mutations, gene fusions, copy number variations, and epigenetic alterations that define sarcoma pathogenesis [2, 4]. Beyond mutation discovery, NGS has empowered researchers to explore the transcriptional dynamics, tumor heterogeneity, and molecular evolution of sarcomas [24, 29].

This review summarizes the applications of NGS in translational sarcoma research, spanning mutation discovery, fusion identification, biomarker and assay development, and the incorporation of computational and multi-omics approaches. It also discusses current limitations, the complementary role of conventional molecular techniques, and the importance of expert interpretation in reference centers when NGS is used in exploratory or clinically oriented settings.

## Mutation and Variant Discovery

Mutation discovery remains one of the most significant contributions of NGS to sarcoma research (Table 1). Early genomic studies identified recurrent mutations in tumor suppressor genes such as *TP53*, *RB1*, and *CDKN2A*/B, and in signaling pathways such as PI3K/*PTEN*/mTOR, which are frequently altered in soft-tissue and bone sarcomas [16, 30]. Nacev et al., [54] demonstrated through large-scale clinical sequencing that sarcomas possess distinct mutational landscapes that vary substantially among subtypes, from translocation-driven tumors like synovial sarcoma to complex karyotype sarcomas such as undifferentiated pleomorphic sarcoma. Recent advances in targeted comprehensive genomic profiling have expanded the catalog of actionable mutations. Bhonde et al., [7] reported novel single-nucleotide variants (SNVs) in rare sarcoma subtypes, while Bajpai et al., [4] linked cellular proliferation and tumor suppression pathways to aggressive disease phenotypes. These discoveries underscore how NGS can uncover both common and subtype-specific driver mutations.

Furthermore, longitudinal sequencing has revealed the dynamic nature of mutational events over time. Studies have shown that recurrent mutations may emerge under therapeutic pressure, emphasizing the adaptive capabilities of sarcomas and the importance of continued genomic monitoring [14]. Collectively, these findings demonstrate that NGS enables a more precise understanding of sarcoma genomics, fostering the identification of novel therapeutic targets and contributing to personalized treatment design.

Importantly, mutation detection alone does not establish clinical actionability. Variant interpretation requires assessment of tumor purity, sequencing depth, germline versus somatic origin when relevant, functional evidence, drug availability, and the level of clinical evidence supporting a matched intervention, ideally according to standardized actionability and reporting frameworks such as ESCAT and ESMO genomic-reporting recommendations [30, 53, 54, 66].

**Table 1:** Most relevant mutations and genomic alterations identified by NGS in sarcomas. Abbreviations: DDLPS, dedifferentiated liposarcoma; DFSP, dermatofibrosarcoma protuberans; EWS, Ewing sarcoma; IMT, inflammatory myofibroblastic tumor; LMS, leiomyosarcoma; LOF, loss-of-function; LPS, liposarcoma; MLPS, myxoid liposarcoma; MPNST, malignant peripheral nerve sheath tumor; PEComa, perivascular epithelioid cell tumor; RMS, rhabdomyosarcoma; UPS, undifferentiated pleomorphic sarcoma; WDLPS, well-differentiated liposarcoma

Gene/ Pathway	Type of alteration	Sarcoma subtypes commonly affected	Approx. frequency (% of Cases)	Functional/Clinical significance	Key references
*TP53*	Missense / truncating mutations, deletions	UPS, LMS, osteosarcoma	35–55%	Central tumor suppressor; mutations linked to genomic instability and poor prognosis	Cote et al., [16], Dorand, [22], Nacev, [54]
*RB1*	Deletions, LOF mutations	Osteosarcoma, UPS, LPS	20–40%	Cell cycle regulation; loss drives uncontrolled proliferation	Bajpai et al., [4], Gounder, [30]
*CDKN2A*/ *CDKN2B*	Homozygous deletions, promoter methylation	Synovial sarcoma, UPS, EWS	15–30%	Loss of G1 checkpoint control; correlated with metastasis	Nacev et al., [54]; Gounder et al., [30]
*MDM2*	Gene amplification	LPS, intimal sarcoma	65–90% in WDLPS/DDLPS	Inhibits *TP53*; diagnostic and targetable alteration	Gounder et al., [30]; [22]
*PIK3CA*	Activating missense mutations	Uterine LMS, angiosarcoma	10–20%	Activates PI3K/AKT/mTOR signaling; potential for PI3K inhibitors	Bajpai et al., [4], Hensley, [35]
*PTEN*	LOF mutations, deletions	LMS, osteosarcoma	10–15%	Tumor suppressor; loss increases mTOR signaling	Hensley et al., [35]; [54]
*ATRX*	Loss-of-function mutations	LMS, angiosarcoma	15–25%	Chromatin remodeling; associated with telomere maintenance (ALT phenotype)	Hensley et al., [35]; [54]
*CDK4*/ *CCND1*	Amplifications	LPS, dedifferentiated sarcoma	60–80%	Drives cell cycle progression; predictive for *CDK4* inhibitor response	Bajpai et al., [4], Gounder, [30]
*NF1*	Inactivating mutations	MPNST, UPS	10–20%	Ras pathway activation; loss promotes MAPK signaling	Dorand et al., [22], Nacev, [54]
*EWSR1*-*FLI1*/*ERG*	Gene fusion	EWS	> 95%	Diagnostic and oncogenic driver fusion	Racanelli et al., [58], Zhu, [76]
*FUS*-*DDIT3*	Gene fusion	MLPS	~ 90%	Defines subtype; transcriptional reprogramming	Aggarwal et al., [1], Lam, [45]
*SYT*-*SSX1*/2/4 (*SS18*-SSX)	Gene fusion	Synovial sarcoma	> 90%	Diagnostic hallmark; epigenetic transcriptional regulator	Lam et al., [45]; [58]
*COL1A1*-*PDGFB*	Gene fusion	DFSP	~ 95%	Constitutive *PDGFB* signaling; imatinib-sensitive	Hu et al., [37], Racanelli, [58]
*ALK*/*ROS1*/ *NTRK1*-3	Gene fusions, rearrangements	IMT, infantile fibrosarcoma, rare soft-tissue sarcomas	5–10% overall; 70–90% in IMT or NTRK+ sarcomas	Oncogenic kinase fusions; highly druggable	Gounder et al., [30], Weiss, [71]
*TERT* promoter	Mutations, rearrangements	Osteosarcoma, LMS	10–20%	Telomerase reactivation; confers immortalization	Bhonde et al., [7], Nacev, [54]
*IDH1*/*IDH2*	Missense mutations	Chondrosarcoma	50–60% (*IDH1*/2 combined)	Alters 2-hydroxyglutarate metabolism; IDH inhibitor target	Dorand et al., [22], Gounder, [30]
*BRAF*/*NRAS*/*KRAS*	Activating mutations	Angiosarcoma, epithelioid sarcoma	5–15%	MAPK pathway activation; potential target for MEK/*BRAF* inhibitors	Dorand et al., [22], Nacev, [54]
*TSC1*/*TSC2*	Inactivating mutations	PEComa, LMS	20–40% (esp. in PEComas)	mTOR pathway activation; predictive of rapalog sensitivity	Bajpai et al., [4], Gounder, [30]
*FAT1*/*NOTCH1*	Mutations, truncations	UPS, LMS	10–15%	Disrupts cell adhesion and Wnt/Notch signaling	Bhonde et al., [7], Grachev, [29]
*BCOR*/*BCORL1*	Mutations, gene fusions	*BCOR* sarcomas, clear cell sarcoma of kidney	50–80% in *BCOR*-rearranged tumors	Defines molecular subtype; transcriptional repression complex	Grachev et al., [29]; [54]
*NAB2*-*STAT6*	Gene fusion	Solitary fibrous tumor	> 90%	EGR1-dependent transcription activation; diagnostic driver fusion	Georgiesh et al., [26], Robinson, [63]
*MYOD1*	Point mutation (most commonly p.L122R)	Spindle cell/sclerosing RMS	30–70%	Oncogenic mutation, aggressive phenotype, transcriptional reprogramming	Kohsaka et al., [44], Rekhi, [59]

### Fusion Gene Identification

Fusion gene identification is among the most important applications of NGS in sarcoma research and diagnostics (Table 2). Several sarcoma subtypes are defined by pathognomonic gene fusions, such as *EWSR1*-*FLI1*/*EWSR1*-*ERG* in Ewing sarcoma, *SS18*-*SSX1*/2/4 in synovial sarcoma, *FUS*-*DDIT3* in MLPS, *COL1A1*-*PDGFB* in dermatofibrosarcoma protuberans, and *NAB2*-*STAT6* in solitary fibrous tumor, PAX3-FOXO1 in alveolar RMS, which play pivotal roles in diagnosis, classification, and, in selected cases, therapeutic stratification [18, 58, 60, 69]. Traditional detection techniques such as FISH and RT-PCR remain valuable, rapid, and widely available tools, but they are limited when the fusion partner is unknown or when multiple diagnostic possibilities must be evaluated simultaneously.

NGS technologies, particularly RNA sequencing and anchored multiplex PCR, have revolutionized fusion gene discovery by enabling unbiased detection of known and novel fusion transcripts [13, 20, 45, 46]. Zhu et al., [76] successfully identified a recurrent *ACTB-FOSB* fusion in pseudomyogenic hemangioendothelioma using targeted RNA sequencing, showcasing NGS’s capability to detect cryptic fusion events. Similarly, Aggarwal et al., [1] validated RNA-based NGS panels that improved classification accuracy in soft-tissue sarcomas.

In addition, genome-wide approaches and hybrid-capture NGS methods have proven effective in detecting structural rearrangements in formalin-fixed, paraffin-embedded (FFPE) samples [37].

The Italian ACC Sarcoma Working Group employed such methods to identify complex rearrangements across numerous subtypes [17, 58]. Beyond classification, fusion discovery through NGS has shed light on fusion-driven oncogenic mechanisms, revealing new potential therapeutic targets.

Thus, RNA-based and targeted NGS approaches have become highly valuable tools for identifying gene fusions in sarcomas, particularly when histology and IHC do not establish a definitive diagnosis [47], when the expected fusion partner is unknown, or when broad molecular characterization is required. However, NGS should not be described as universally replacing FISH, RT-PCR, or IHC. Rather, its diagnostic and translational value depends on the clinical question, tissue quality, assay design, bioinformatic validation, and expert interpretation within multidisciplinary sarcoma and molecular tumor boards [53, 66, 69].

For known recurrent rearrangements in a well-defined clinicopathological context, FISH or RT-PCR may remain faster, cheaper, and sufficient; NGS adds most value when the differential diagnosis is broad, the fusion partner is unexpected, the sample is limited, or multiple alteration classes must be interrogated simultaneously [37, 45, 46, 58, 60, 69].

**Table 2:** Most relevant fusion genes identified by NGS in sarcomas. Abbreviations: DFSP, dermatofibrosarcoma protuberans; EWS, Ewing sarcoma; IMT, inflammatory myofibroblastic tumor; LPS, liposarcoma; MLPS, myxoid liposarcoma; RMS, rhabdomyosarcoma; STS, soft tissue sarcoma

Fusion gene	Sarcoma subtype(s)	Approx. frequency	Functional / Clinical significance	Key references
*EWSR1*–*FLI1* / *EWSR1*–*ERG*	EWS	> 95%	Pathognomonic; major diagnostic criterion; transcriptional reprogramming driver	Racanelli et al., [58], Zhu, [76]
*SS18*–*SSX1*/*SSX2* (*SYT*–SSX)	Synovial sarcoma	> 90%	Defines subtype; chromatin remodeling; central oncogenic driver	Lam et al., [45], Racanelli, [58]
*FUS*–*DDIT3*	MLPS	~ 90%	Diagnostic hallmark; alters adipocytic differentiation; prognostic relevance	Lam et al., [45]; [1]
*COL1A1*–*PDGFB*	DFSP	~ 95%	Constitutive *PDGFB* signaling; imatinib-sensitive targetable fusion	Hu et al., [37], Racanelli, [58]
*NTRK1*/2/3 fusions (various partners)	Infantile fibrosarcoma; rare STS	70–90% in NTRK+ sarcomas	Oncogenic kinase activation; highly actionable with TRK inhibitors	Gounder et al., [30], Weiss, [71]
*ALK* rearrangements (e.g., TPM3–*ALK*)	IMT	50–60%	Tyrosine kinase activation; *ALK* inhibitor–sensitive	Weiss et al., [71]; [30]
*ROS1* fusions	IMT; rare STS	~ 10%	Kinase activation; targetable with *ROS1* inhibitors	Weiss et al., [71]; [30]
*BCOR*–*CCNB3*	*BCOR*–rearranged sarcoma	50–80%	Defines a molecular subtype; transcriptional repression complex dysregulation	Grachev et al., [29], Nacev, [54]
*PAX3-FOXO1*	alveolar RMS	60–70%	primary molecular driver	Linardic, [48]

### Tumor Heterogeneity and Evolution

Sarcomas exhibit extensive heterogeneity both within individual tumors (intra-tumoral) and across patients (inter-tumoral), which contributes to variable treatment responses and disease progression. NGS has emerged as a critical tool for characterizing this heterogeneity and mapping clonal evolution over time [4, 15, 24].

Single-cell sequencing and multi-region sampling have provided unprecedented insight into the clonal composition of sarcomas. (Grachev et al., [29], [28]) used transcriptomic analyses to detect intra- and transchromosomal fusions that arise during tumor evolution, identifying specific clonal lineages associated with metastasis and therapy resistance. Eisenhardt et al., [24] applied circulating tumor DNA (ctDNA) genotyping to monitor synovial sarcoma dynamics, demonstrating how NGS can capture real-time tumor evolution and resistance mechanisms.

Longitudinal sequencing of pediatric and adult sarcomas has revealed that mutations in *TP53*, *MDM2*, and *ATRX* often evolve under selective pressure from treatment, driving clonal diversification [14]. These findings highlight the adaptability of sarcoma genomes and underscore the need for integrative temporal profiling to optimize treatment strategies.

In essence, NGS enables a multidimensional understanding of sarcoma heterogeneity—spanning genetic, spatial, and temporal dimensions—laying the groundwork for more dynamic therapeutic approaches.

### Biomarker and Target Discovery

The discovery of predictive and prognostic biomarkers is a cornerstone of modern oncology, and NGS has accelerated this process in sarcoma research. By analyzing whole-exome or targeted sequencing data, researchers have identified molecular alterations that correlate with clinical outcomes and drug response [35, 49, 68].

Hensley et al., [35] used NGS to define the genomic landscape of uterine sarcomas, identifying actionable alterations in *PIK3CA*, *PTEN*, and *ATRX*. Similarly, Dufresne et al., [23] highlighted how biology-driven therapeutic selection can improve outcomes in aggressive sarcomas, emphasizing the potential of genomics-informed treatment.

NGS also facilitates the identification of immune-related biomarkers. Studies using RNA-seq have characterized immune infiltration and tumor microenvironmental features that predict response to immunotherapy [24]. Moreover, computational interpretation of NGS data through machine learning approaches has allowed the discovery of non-obvious associations between gene expression signatures and therapeutic vulnerability [29].

In this regard, NGS has also revolutionized epigenomic research by enabling genome-wide DNA methylation profiling, complementing genomic and transcriptomic analyses. Whole-genome bisulfite sequencing (WGBS) and reduced representation bisulfite sequencing (RRBS) via NGS platforms reveal dynamic methylation patterns at CpG sites, uncovering sarcoma-specific epigenetic landscapes that drive heterogeneity and clonal evolution [6, 51].

NGS-based methylation studies highlight sarcoma subtypes’ distinct profiles: Ewing sarcoma exhibits hypomethylation at developmental enhancers, while synovial sarcoma shows hypermethylation of tumor suppressor promoters. Reduced representation bisulfite sequencing identifies differentially methylated regions (DMRs) linking methylation to fusion gene expression, such as *SS18*-SSX in synovial sarcomas, influencing chromatin accessibility. These patterns correlate with clonal dynamics, where methylation entropy tracks tumor evolution in leiomyosarcomas [10, 43, 51, 52

Integrating methylation arrays with NGS multi-omics reveals pan-sarcoma signatures; for instance, MethMarkerDB compiles 5.4 million DMRs from 658 WGBS datasets, pinpointing sarcoma-enriched loci for prognostic modeling. In undifferentiated sarcomas, methylation classifiers distinguish CIC/Bcor-altered entities from Ewing, aiding subtype deconvolution. Single-cell NGS epigenomics further dissects intratumor heterogeneity, exposing methylation gradients in mesenchymal progenitors [6, 52, 77].

Machine learning on NGS methylation data deciphers regulatory networks, predicting variant impacts on epigenomic states. Challenges include bisulfite conversion biases, addressed by long-read NGS for phase-resolved methylation. Future integration with spatial transcriptomics promises to map methylation in sarcoma microenvironments, advancing discovery research [10, 11, 38].

### Assay Development and Validation

In addition to discovery, NGS technologies have facilitated the development and validation of novel molecular assays. These assays enable sensitive, multiplexed detection of fusions, mutations, and other variants for both research and diagnostic use. Lam et al., [45] pioneered the use of anchored multiplex PCR–NGS for fusion detection, achieving superior sensitivity compared to conventional methods. Lanic et al., [46] extended this work by creating a ligation-dependent multiplex RT-PCR NGS assay capable of detecting diverse fusion events across sarcoma subtypes.

Hu et al., [37] developed and validated an RNA sequencing panel optimized for FFPE samples, overcoming degradation issues common in archival tissue. Similarly, Jeyaraman et al., [40] designed custom gene panels for osteosarcoma and Ewing sarcoma, enabling focused mutation profiling and improving assay adaptability for low-quality samples.

These efforts have enhanced assay reproducibility, sensitivity, and integration into diagnostic workflows.

As NGS technologies continue to evolve, long-read sequencing and highly sensitive digital assays may improve analytical resolution and help address specific diagnostic questions in selected sarcoma settings, although their routine implementation requires technical validation and evidence of added clinical value [40, 56, 66].

Nevertheless, assay performance is strongly influenced by pre-analytical variables, nucleic-acid quality, panel design, fusion-calling algorithms, validation cohorts, and reporting standards. Therefore, diagnostic implementation requires orthogonal confirmation when appropriate and interpretation by molecular pathologists familiar with sarcoma-specific entities [37, 45, 46, 56, 66].

### Multi-Omics Integration and Computational Approaches

An emerging direction in modern sarcoma research is the integration of multi-omics data—combining genomic, transcriptomic, proteomic, and epigenomic information—to provide a holistic view of tumor biology and offer new opportunities for personalized medicine. However, the analysis and interpretation of high-throughput sequencing data pose significant challenges due to the high dimensionality, complexity, and heterogeneity [25, 42].

Computational methods leveraging statistical and machine learning (ML) approaches are becoming indispensable tools for addressing these challenges and further advancing precision oncology. An effort in this direction has been made by The Cancer Genome Atlas Research Network, which provided a comprehensive and integrated characterization of 206 adult soft tissue sarcomas based on a multi-platform genomic landscape [9].

Approaches for multi-omics data integration have been employed to identify mutational signatures, predict clinical outcomes [12], and monitor tumor dynamics [24]. These algorithms have proven particularly valuable for sarcoma classification and target identification [42, 65].

Moreover, the use of NGS-based approaches has led to a substantial reclassification of histopathological diagnoses, as reported in a recent study [55].

ML approaches are an emerging key component of such integrative approaches. ML models can learn complex patterns from high-dimensional multimodal data to predict various functional outcomes. The combined use of ML and NGS has facilitated the identification of novel prognostic and diagnostic biomarkers [64, 67] and improved the classification of distinct sarcoma subtypes [25]. Recently, a multi-algorithm ML framework identified a G protein–coupled receptor (GPR) signature with diagnostic and clinical relevance in soft tissue sarcomas [70]. In addition, first attempts have been made to derive ML models by combining biological and chemical data and predict candidate compounds for sarcoma treatment via drug repurposing [3] or activity predictions based on cell transcriptomic and compound data [72].

Thus, the integration of NGS with systems and chemical biology, encompassing both classical statistical approaches and ML models, adds a new dimension to research in complex cancer genomics.

### Translational Implications and Clinical Integration

The translation of NGS discoveries into clinical practice marks one of the most promising but also most complex developments in sarcoma research [8] (Table 3). Clinical sequencing efforts, such as those reported by Gounder et al., [30], have demonstrated how genomic profiling can inform patient management by identifying actionable targets, supporting molecular classification, and guiding enrollment into precision-oncology trials. However, the clinical relevance of NGS findings varies substantially across sarcoma subtypes and should be interpreted in the context of validated biomarkers, available therapies, ESCAT levels of actionability, and clinical-trial access [53, 66]. Molecular tumor boards and sarcoma reference centers increasingly incorporate NGS results to recommend personalized treatment options, bridging the gap between research and clinical care [5]. Referral to expert centers is particularly important for rare sarcoma entities, diagnostically ambiguous tumors, suspected translocation-driven sarcomas, and advanced or refractory disease in which broad molecular profiling may identify tumor-agnostic biomarkers, actionable kinase fusions, or eligibility for molecularly matched trials. In routine practice, NGS should be integrated with morphology, IHC, FISH, RT-PCR [73], methylation profiling when appropriate, and clinical information rather than interpreted as an isolated result [17, 53, 66, 69].

In practical terms, broad NGS is most informative in diagnostically challenging sarcomas, tumors with suspected but unconfirmed gene fusions, advanced or refractory disease, and rare entities in which molecular findings may refine classification or identify clinical-trial eligibility [73]. Examples include RNA-based fusion testing in Ewing sarcoma-like tumors, synovial sarcoma, MLPS, IMT, infantile fibrosarcoma and other NTRK-rearranged sarcomas, as well as targeted profiling for clinically relevant alterations when matched therapies or molecularly driven trials are available [30, 53, 55, 66, 69].

For example, patients harboring NTRK or *ALK* fusions identified by NGS have benefited from targeted kinase inhibitors, underscoring the tangible clinical value of genomic data [71]. NGS is a valuable tool for detecting mutations that can be targeted by emerging therapies, by identifying actionable alterations, molecular profiling can help expand therapeutic options and potentially improve survival outcomes [22] (Table 3).

**Table 3:** Overview of NGS-Based Molecular Profiling Studies and Clinical Utility in Advanced Sarcomas. Abbreviations: GIST, gastrointestinal stromal tumor; NGS, next-generation sequencing; N/R, not reported. * The table reports the clinical benefits, defined as stable disease for more than 6 months

Study	Tumor type	No. of samples	Tumors with ≥ 1 genomic alteration	% of patients with clinically actionable mutations	Most frequent alterations	Patients with NGS-influenced treatment	Clinical benefit observed*
Gusho et al., [33]	Non-GIST sarcomas (116 soft tissue, 20 bone)	136	89.7%	47.1%	*TP53*, *CDKN2A*/B, *RB1*, *CDKN2A*	12	3/12
Boddu et al., [8]	Non-GIST sarcomas (94 soft tissue, 20 bone)	114	96.7%	49.1%	*TP53*, *CDKN2A*/B, *CDK4*/*MDM2*, *ATRX*, *RB1*.	15	4/15
Groisberg et al., [31]	Advanced sarcomas	102	93%	N/R	*TP53*, *CDK4*, *MDM2*, *RB1*, *CDKN2A*/B.	16	8/16
Gupta et al., [32]	Advanced sarcomas	108	N/R	24.4%	*N/R*	16	4/16
Jin et al., [41]	Soft Tissue Sarcomas	65	95.4%	36.9%	*TP53*, *MDM2*, *CDK4*, *EWSR1*, KDR, *NF1*.	4	2/4
T Diab et al., [21]	Non-GIST sarcomas (55 soft tissue, 23 bone)	78	87%	42%	*CDK4*, *PIK3CA*, *NF1*, *ALK*	16	6/16

Although NGS-based molecular profiling may identify additional therapeutic opportunities with the potential to improve survival, available data in this clinical setting remain limited, with even fewer studies systematically evaluating the clinical utility of these approaches. Moreover, the predominantly retrospective design of the published studies and the frequent implementation of molecular profiling at advanced stages of disease represent important limitations that may restrict the clinical benefit of genomically guided therapeutic interventions.

Moreover, liquid biopsy approaches leveraging ctDNA sequencing are enabling non-invasive tumor monitoring, capturing the genomic evolution of sarcomas in real time [24].

Liquid biopsy approaches based on ctDNA sequencing may provide a complementary strategy for non-invasive monitoring in selected sarcoma contexts, particularly when longitudinal tissue sampling is not feasible. However, their sensitivity varies by tumor burden, histotype, shedding rate, disease site, and assay design, and their routine use in sarcoma still requires further validation [14, 15, 24, 66].

Overall, the role of NGS in sarcoma research and selected clinical applications is expanding because it enables comprehensive genomic characterization, fusion discovery, investigation of tumor heterogeneity, biomarker and assay development. At the same time, technical, analytical, economic, and interpretive challenges remain, and the clinical impact of NGS depends on subtype-specific evidence, access to matched therapies, and multidisciplinary interpretation (Supplementary Table 1).

### Challenges and Future Directions

Despite its transformative potential, several challenges limit the full realization of NGS in sarcoma research and practice. Technical issues such as degraded RNA in FFPE samples, low tumor purity, insufficient nucleic-acid input, and difficulty detecting low-frequency variants persist [56]. Bioinformatics complexity, including data storage, normalization, variant classification, fusion calling, and clinically meaningful reporting, continues to be a major bottleneck [39, 66]. These limitations should be considered alongside the strengths of established complementary methods: FISH and RT-PCR can be rapid and cost-effective for selected known rearrangements, IHC remains essential for lineage and surrogate marker assessment, and methylation classifiers may support diagnosis in selected ambiguous sarcomas.

Additionally, the heterogeneity and rarity of sarcomas complicate statistical validation of genomic findings. Large-scale, multi-institutional collaborations are therefore essential to achieve sufficient cohort sizes for robust analysis. Initiatives such as the CCCLMU Molecular Tumor Board have demonstrated the success of collaborative genomic data sharing [5].

Future directions include integrating single-cell and spatial transcriptomics with NGS to uncover interactions between tumor cells and their microenvironment [4, 28] (Table 4). Combining genomics with proteomics, metabolomics, and functional models could also uncover dependencies that drive sarcoma growth and resistance. Ultimately, standardized computational pipelines, validated reporting frameworks, international data sharing, and open-access genomic repositories will be key to translating NGS data into reproducible biological and clinical insights [53, 66].

**Table 4:** Future directions for advancing NGS research applications in sarcoma. Abbreviations: ML, machine learning; NGS, next-generation sequencing

Open questions	Relevance
**How can single-cell and spatial transcriptomics be integrated with NGS to better understand sarcoma heterogeneity?**	Integration could reveal new insights into tumor evolution, microenvironment, and resistance mechanisms.
**What are the most effective computational and machine learning approaches for analyzing complex NGS data in sarcoma research?**	Improved tools are needed to interpret large, multi-dimensional datasets and identify novel patterns.
**How can NGS-based research be expanded to cover rare and understudied sarcoma subtypes?**	Broader research will help uncover unique genetic drivers and inform future translational studies.

Future efforts combining NGS-based biomarker discovery with innovative therapeutic platforms, including nanomedicine, may open new avenues for precision treatment in soft tissue sarcomas. However, these approaches remain largely preclinical and should be presented as emerging research directions rather than established clinical strategies. In this context, NGS-derived biomarkers may support the rational design of targeted drug-delivery systems [36], including nanoparticle-based platforms functionalized to recognize tumor-associated alterations or microenvironmental features, but such strategies require rigorous validation of target specificity, biodistribution, efficacy, and off-target toxicity before clinical translation. However, as reported by [75] the intrinsic heterogeneity of STSs poses significant challenges to the development of universally effective nanoparticle-based therapeutic strategies, highlighting the need for subtype-specific approaches [50].

Mutations and molecular vulnerabilities identified through omics analyses must be validated in appropriate preclinical models to elucidate their biological functions and their implications for therapeutic response. In vivo models remain important because they can capture tumor-host interactions, vascularization, immune components, and systemic pharmacology more effectively than simplified in vitro systems. The choice of model should be driven by the biological question, sarcoma subtype, genetic alteration, and intended translational endpoint [19, 34, 60–62].

Zebrafish models are highly amenable to genetic manipulation and can provide a rapid, cost-effective in vivo platform for investigating oncogenic mechanisms, developmental pathways, tumor dissemination, and drug response in sarcoma research. Their optical transparency, fecundity, and suitability for high-throughput screening represent important advantages, while evolutionary distance from humans, differences in immune maturation, and limitations in modeling some aspects of the mammalian tumor microenvironment should also be acknowledged ([34, 60] b, [27]).

The partial genomic redundancy resulting from the teleost genome duplication can facilitate the study of genetic interactions and modifier genes through the generation of compound mutants. Recent genome-editing approaches, including optimized CRISPR-based methods and emerging prime-editing strategies, may further improve the precision of disease-specific zebrafish model development. Once validated, these models can complement murine, organoid, patient-derived xenograft, and cell-line systems as platforms for functional genomics and drug screening in rare sarcoma subtypes ([34, 60] b).

## Conclusions

Next-generation sequencing has substantially advanced sarcoma research by enabling the detection of genomic and transcriptomic alterations that contribute to the biology, classification, and clinical investigation of these rare malignancies. Its major contributions include mutation and variant discovery, fusion detection, biomarker development, assay validation, and integration with multi-omics and computational approaches. Nevertheless, NGS should be considered a powerful complementary technology rather than an all-purpose replacement for histopathology, IHC, FISH, RT-PCR, or other established diagnostic methods.

Future progress will depend on careful integration of NGS with single-cell sequencing, spatial transcriptomics, methylation profiling, functional models, artificial intelligence, and prospective clinical studies. For patients with sarcoma, the greatest impact is likely to occur when molecular data are generated using validated assays, interpreted by expert multidisciplinary teams, and linked to subtype-specific evidence, actionable biomarkers, and clinical-trial opportunities.

A balanced implementation of NGS may therefore strengthen both biological discovery and precision oncology while avoiding overinterpretation of exploratory findings. 

Highlights the diagnostic value of broad RNA-based fusion detection for improving molecular classification of soft tissue sarcomas. 

## Key Reference

- Aggarwal et al. (2025). RNA-based fusion NGS panel testing for the improved classification of soft tissue sarcomas.
  - ○ Highlights the diagnostic value of broad RNA-based fusion detection for improving molecular classification of soft tissue sarcomas.
- Miettinen et al. (2024). Assessment of the utility of the sarcoma DNA methylation classifier in surgical pathology.
  - ○ Supports the role of genome-wide methylation-based classifiers in diagnostically challenging sarcomas.
- Gounder et al. (2022). Clinical genomic profiling in the management of patients with soft tissue and bone sarcoma.
  - ○ Provides clinical genomic profiling data relevant to management and molecular stratification of sarcoma patients.
- Wallander et al. (2023). Sarcoma care in the era of precision medicine.
  - ○ Provides a precision-medicine framework for integrating molecular diagnostics, prognostic markers, and therapeutic implications in sarcoma care.
- Mosele et al. (2024) and van de Haar et al. (2024). ESMO precision-medicine and genomic-reporting recommendations.
  - ○ Support cautious interpretation, standardized reporting, and clinically meaningful actionability assessment of NGS findings.

## Electronic Supplementary Material

Below is the link to the electronic supplementary material.

Supplementary Material 1 (26.0 KB)

Supplementary Material 2 (14.9 KB )

## Data Availability

The datasets generated and/or analysed during the current study are available from the corresponding author on reasonable request.

## Abbreviations

DFSP: dermatofibrosarcoma protuberans
DDLPS: dedifferentiated liposarcoma
DMR: differentially methylated region
ESMO: European Society for Medical Oncology
ESCAT: ESMO Scale for Clinical Actionability of molecular Targets
EWS: Ewing sarcoma
FFPE: formalin-fixed paraffin-embedded
FISH: fluorescence in situ hybridization
GIST: gastrointestinal stromal tumor
GPR: G protein-coupled receptor
IMT: inflammatory myofibroblastic tumor
IHC: immunohistochemistry
LMS: leiomyosarcoma
LOF: loss-of-function
LPS: liposarcoma
ML: machine learning
MPNST: malignant peripheral nerve sheath tumor
NGS: next-generation sequencing
PEComa: perivascular epithelioid cell tumor
RMS: rhabdomyosarcoma
RRBS: reduced representation bisulfite sequencing
RT-PCR: reverse-transcription polymerase chain reaction
SNV: single-nucleotide variant
STS: soft tissue sarcoma
UPS: undifferentiated pleomorphic sarcoma
WDLPS: well-differentiated liposarcoma
WGBS: whole-genome bisulfite sequencing

## References

[1] Aggarwal S, Rastogi S, Gowda H, Shri P, Bahl C, Natarajan A, Ganesh J, Farhin S, Coral K, Rehman S. RNA-based fusion NGS panel testing for the improved classification of soft tissue sarcomas: A retrospective analysis. J Clin Oncol. 2025. 10.1200/jco.2025.43.16_suppl.11541.40138611

[2] Aktiq M, Balan J, Blackburn P, Gross J, Voss J, Jin L, Fadra N, Davila J, Pitel B, Terra S, Minn K, Jackson R, Hofich C, Willkomm K, Peterson B, Clausen S, Rumilla K, Gupta S, Lo Y, Halling K. SARCP, a clinical next generation sequencing assay for the detection of gene fusions in sarcomas: A description of the first 652 cases. J Mol Diagnostics: JMD. 2024. 10.1016/j.jmoldx.2024.10.004. 39521244

[3] Baek B, Jang E, Park S, Park SH, Williams DR, Jung DW, Lee H. Integrated drug response prediction models pinpoint repurposed drugs with effectiveness against rhabdomyosarcoma. PLoS ONE. 2024;19:e0295629. 10.1371/journal.pone.0295629. 38277404 PMC10817174

[4] Bajpai J, Kishan D, Basavalingegowda M, Hariramani K, Ramesh A, Iyer S, Dasu K, Jadhav B, Prajapati S, D’Souza A, Singh P, Vasudevan A, Bharde A, Uttarwar M, Khandare J, Shafi G. Cellular proliferation and tumor suppression pathways as potential targets in clinically aggressive sarcomas. J Clin Oncol. 2025. 10.1200/jco.2025.43.16_suppl.e23551.41115255

[5] Berclaz L, Burkhard-Meier A, Lange P, Di Gioia D, Schmidt M, Knösel T, Klauschen F, von Bergwelt-Baildon M, Heinemann V, Greif P, Westphalen C, Heinrich K, Lindner L. Implementing precision oncology for sarcoma patients: The CCCLMU Molecular Tumor Board experience. J Cancer Res Clin Oncol. 2023;149:13973–83. 10.1007/s00432-023-05179-y. 37542550 PMC10590320

[6] Bhattacharya S, Makkar H, Meena JP, Gupta AK, Seth R. Epigenetic paradigm of DNA methylation for understanding the pathophysiology, diagnostics, and therapeutics in sarcomas. Epigenomics. 2025;17(16):1213–24. 10.1080/17501911.2025.2563500. 40971712 PMC12562784

[7] Bhonde M, Bose C, Gupta V, Tiwari P, D’Souza A, Kad T, Prajapati S, Jadhav B, Hariramani K, Basavalingegowda M, Mobeen F, Rani A, Mehra V, Kumaran M, Haldar S, Saha R, Mehta P, Uttarwar M, Khandare J, Shafi G. Identification of novel actionable SNVs using targeted comprehensive genome profiling in patients with sarcoma. J Clin Oncol. 2023. 10.1200/jco.2023.41.16_suppl.e23520.

[8] Boddu S, Walko CM, Bienasz S, Bui MM, Henderson-Jackson E, Naghavi AO, Brohl AS. Clinical utility of genomic profiling in the treatment of advanced sarcomas: a single-center experience. JCO Precision Oncol. 2018;2:1–8. 10.1200/PO.18.00096 35135155

[9] Cancer Genome Atlas Research Network. Comprehensive and integrated genomic characterization of adult soft tissue sarcomas. Cell. 2017;171(4):950–e96528. 10.1016/j.cell.2017.10.014. 29100075 PMC5693358

[10] Chantre-Justino M, Meohas W. The DNA methylation landscape of musculoskeletal sarcomas. Explor Target Anti-Tumor Therapy. 2025;6:1002319. 10.37349/etat.2025.1002319. PMC12123220 40452888

[11] Chen Y, Zhu R, Chen M, Guo W, Yang X, Xu XJ, Zhu L. Prognostic value of a three-DNA methylation biomarker in patients with soft tissue sarcoma. J Oncol. 2020;2020:8106212. 10.1155/2020/8106212. 32508922 PMC7245661

[12] Chibon F, Lagarde P, Salas S, et al. Validated prediction of clinical outcome in sarcomas and multiple types of cancer on the basis of a gene expression signature related to genome complexity. Nat Med. 2010;16:781–7. 10.1038/nm.2174. 20581836

[13] Cheng Y, Meyer A, Jakubowski M, Keenan S, Brock J, Azzato E, Weindel M, Farkas D, Rubin B. Gene fusion identification using anchor-based multiplex PCR and next-generation sequencing. J Appl Lab Med. 2021. 10.1093/jalm/jfaa230. 33537766

[14] Comeau H, De La Vega L, Church A, Maese L, Pinto N, Macy M, Applebaum M, Sabnis A, Kim A, Colace S, Cruz F, Dubois S, Collins N, Janeway K. Abstract 2862: Evolution of clinically relevant variants in advanced pediatric sarcomas. Cancer Res. 2024. 10.1158/1538-7445.am2024-2862.

[15] Connell M, Gazdová J, Beck K, Srivastava S, Harewood L, Stewart J, Huebschmann D, Stenzinger A, Glimm H, Heilig C, Fröhling S, Gonzalez D. Detection of structural variants in circulating cell-free DNA from sarcoma patients using next generation sequencing. Cancers. 2020;12. 10.3390/cancers12123627. PMC7761870 33287361

[16] Cote G, He J, Choy E. Next-generation sequencing for patients with sarcoma: A single-center experience. Oncologist. 2018;23(2):234–42. 10.1634/theoncologist.2017-0290. 28860410 PMC5813739

[17] D’Ambrosio L, Sbaraglia M, Merlini A, et al. Extended molecular profiling in mesenchymal tumors: a consensus paper from the Italian Sarcoma Group. Crit Rev Oncol Hematol. 2025;216:104960. 10.1016/j.critrevonc.2025.104960. 40998028

[18] Dancsok AR, Asleh-Aburaya K, Nielsen TO. Advances in sarcoma diagnostics and treatment. Oncotarget. 2017;8(4):7068–93. 10.18632/oncotarget.12548. 27732970 PMC5351692

[19] De Vita A, Recine F, Miserocchi G, et al. The potential role of the extracellular matrix in the activity of trabectedin in UPS and L-sarcoma: evidences from a patient-derived primary culture case series in tridimensional and zebrafish models. J Exp Clin Cancer Res. 2021;40(1):165. 10.1186/s13046-021-01963-1 . PMID: 33975637; PMCID: PMC8111914. 33975637 PMC8111914

[20] De Vita A, Ferrari A, Miserocchi G, et al. Identification of a novel RAB3IP-HMGA2 fusion transcript in an adult head and neck rhabdomyosarcoma. Oral Dis. 2022;28(7):2052–4. 10.1111/odi.14036. 34592033

[21] Diab T, Tarhini A, Jaber G, Raffoul C, Zeineddine N, Kreidieh L, Assi HI. Real-World Implementation of Next-Generation Sequencing in Sarcoma: Molecular Insights and Therapeutic Outcomes. Med Sci. 2026;14(1):46. 10.3390/medsci14010046 PMC12821713 41562936

[22] Dorand R, Robinson M, King L, Schremp E, Miranda A, Colazo J, Park B, Luo L, Shu X, Pal T, Friedman D, Davis E. Next generation sequencing reveals targetable mutations in multiple sarcoma histologies. J Clin Oncol. 2023. 10.1200/jco.2023.41.16_suppl.11548.

[23] Dufresne A, Brahmi M, Karanian M, Blay J. Using biology to guide the treatment of sarcomas and aggressive connective-tissue tumours. Nat Reviews Clin Oncol. 2018;15:443–58. 10.1038/s41571-018-0012-4. 29666441

[24] Eisenhardt A, Brugger Z, Lausch U, Kiefer J, Zeller J, Runkel A, Schmid A, Bronsert P, Wehrle J, Leithner A, Liegl-Atzwanger B, Giunta R, Eisenhardt S, Braig D. Genotyping of circulating free DNA enables monitoring of tumor dynamics in synovial sarcomas. Cancers. 2022;14. 10.3390/cancers14092078. PMC9105697 35565213

[25] Gala P, Pandloskar Y, Godbole S, Hakim F, Kanani P, Kurup L. Classification of sarcoma based on genomic data using machine learning models. Procedia Comput Sci. 2025;252:317–30. 10.1016/j.procs.2024.12.034.

[26] Georgiesh T, Namløs HM, Sharma N, et al. Clinical and molecular implications of *NAB2*-*STAT6* fusion variants in solitary fibrous tumour. Pathology. 2021;53(6):713–9. 10.1016/j.pathol.2020.11.010. 33745702

[27] Giusti V, Miserocchi G, Sbanchi G, Pannella M, Hattinger CM, Cesari M, Fantoni L, Guerrieri AN, Bellotti C, De Vita A, Spadazzi C, Donati DM, Torsello M, Lucarelli E, Ibrahim T, Mercatali L. Xenografting Human Musculoskeletal Sarcomas in Mice, Chick Embryo, and Zebrafish: How to Boost Translational Research. Biomedicines. 2024;12(8):1921. 10.3390/biomedicines12081921 . PMID: 39200384; PMCID: PMC11352184. 39200384 PMC11352184

[28] Grachev D, Ivanov D, Baranov O, Kushnarev V, Clemons M, Yong S, Kotlov N, Chernyshov K, Bagaev A, Fowler N, Conley A, Coté G, Chawla S. Detecting hotspots of intra- and transchromosomal fusions in liposarcomas by RNA sequencing. J Clin Oncol. 2025. 10.1200/jco.2025.43.16_suppl.11561.

[29] Grachev D, Ivanov D, Kushnarev V, Yong S, Kotlov N, Chernyshov K, Bagaev A, Fowler N, Conley A, Coté G, Chawla S. Transcriptomic analysis of novel tumor suppressor gene fusions in bone sarcomas. J Clin Oncol. 2024. 10.1200/jco.2024.42.16_suppl.11523.

[30] Gounder M, Agaram N, Trabucco S, Robinson V, Ferraro R, Millis S, Krishnan A, Lee J, Attia S, Abida W, Drilon A, Chi P, Angelo S, Dickson M, Keohan M, Kelly C, Agulnik M, Chawla S, Choy E, Tap W. Clinical genomic profiling in the management of patients with soft tissue and bone sarcoma. Nat Commun. 2022;13:30496. 10.1038/s41467-022-30496-0. PMC9200814 35705558

[31] Groisberg R, Hong DS, Holla V, Janku F, Piha-Paul S, Ravi V, Subbiah V. Clinical genomic profiling to identify actionable alterations for investigational therapies in patients with diverse sarcomas. Oncotarget. 2017;8(24):39254. 28424409 10.18632/oncotarget.16845 PMC5503611

[32] Gupta K, Grimison P, Fernandez K, Choi V, Bhadri V. Real-world clinical utility of tumor next-generation sequencing in adolescent and young adult patients with sarcoma. JCO Precision Oncol. 2025;9:e2500228. 10.1200/po-25-00228. 40601894

[33] Gusho CA, Weiss MC, Lee L, Gitelis S, Blank AT, Wang D, Batus M. The clinical utility of next-generation sequencing for bone and soft tissue sarcoma. Acta Oncol. 2022;61(1):38–44. 34686105 10.1080/0284186X.2021.1992009

[34] Hayes MN, Langenau DM. Discovering novel oncogenic pathways and new therapies using zebrafish models of sarcoma. Methods Cell Biol. 2017;138:525–61. 10.1016/bs.mcb.2016.11.011. 28129857

[35] Hensley M, Chavan S, Solit D, Murali R, Soslow R, Chiang S, Jungbluth A, Bandlamudi C, Srinivasan P, Tap W, Rosenbaum E, Taylor B, Donoghue M, Hyman D. Genomic landscape of uterine sarcomas defined through prospective clinical sequencing. Clin Cancer Res. 2020;26:3881–8. 10.1158/1078-0432.ccr-19-3959. 32299819 PMC7367750

[36] Hristova-Panusheva K, Xenodochidis C, Georgieva M, Krasteva N. Nanoparticle-Mediated Drug Delivery Systems for Precision Targeting in Oncology. Pharmaceuticals (Basel). 2024;17(6):677. 10.3390/ph17060677 . PMID: 38931344; PMCID: PMC11206252. 38931344 PMC11206252

[37] Hu W, Yuan L, Zhang X, Ni Y, Hong D, Wang Z, Li X, Ling Y, Zhang C, Deng W, Tian M, Ding R, Song C, Li J, Zhang X. Development and validation of an RNA sequencing panel for gene fusions in soft tissue sarcoma. Cancer Sci. 2022;113:1843–54. 10.1111/cas.15317. 35238118 PMC9128172

[38] Ibrahim J, Peeters M, Van Camp G, de Op K. Methylation biomarkers for early cancer detection and diagnosis: Current and future perspectives. Eur J Cancer. 2023;178:91–113. 10.1016/j.ejca.2022.10.015. 36427394

[39] Itkin B, Al-Sayegh H, Deshpande P, Abdou D, Pullanhi A, Sawaya Z, Zadjali A, Haddabi A. Influence of next generation sequencing on sarcomas management in the real-world setting in Oman: Retrospective single institution analysis. ESMO Open. 2024. 10.1016/j.esmoop.2024.102524.

[40] Jeyaraman N, Jeyaraman M, Subramanian P, Ramasubramanian S, Balaji S, Muthu S, Rajendran R, Gangadaran P. Advancements in bone malignancy research through next-generation sequencing focussed on osteosarcoma, chondrosarcoma, and Ewing sarcoma. Pathol Res Pract. 2025;269:155908. 10.1016/j.prp.2025.155908. 40086338

[41] Jin G, Wang C, Jia D, Qian W, Yin C, Wang D, Zheng A. Next generation sequencing reveals pathogenic and actionable genetic alterations of soft tissue sarcoma in Chinese patients: a single center experience. Technol Cancer Res Treat. 2021;20:15330338211068964. 34939467 10.1177/15330338211068964 PMC8721396

[42] Kim J, Kim JH, Kang HG, Park SY, Yu JY, Lee EY, Oh SE, Kim YH, Yun T, Park C, Cho SY, You HJ. Integrated molecular characterization of adult soft tissue sarcoma for therapeutic targets. BMC Med Genet. 2018;19(Suppl 1):216. 10.1186/s12881-018-0722-6. 30598078 PMC6311917

[43] Khan AA, Kumar RN, Chakma S, Das S. Sarcoma diagnosis by DNA methylation classifier: A systematic review, current status and future prospects. Pathol Res Pract. 2024;263:155634. 10.1016/j.prp.2024.155634. 39383738

[44] Kohsaka S, Shukla N, Ameur N, et al. A recurrent neomorphic mutation in *MYOD1* defines a clinically aggressive subset of embryonal rhabdomyosarcoma associated with PI3K-AKT pathway mutations. Nat Genet. 2014;46(6):595–600. 10.1038/ng.2969. 24793135 PMC4231202

[45] Lam S, Cleton-Jansen A, Cleven A, Ruano D, van Wezel T, Szuhai K, Bovée J. Molecular analysis of gene fusions in bone and soft tissue tumors by anchored multiplex PCR-based targeted next-generation sequencing. J Mol Diagn. 2018;20(5):653–63. 10.1016/j.jmoldx.2018.05.007. 30139549

[46] Lanic M, Loarer L, Rainville V, Sater V, Viennot M, Beaussire L, Viailly P, Angot É, Hostein I, Jardin F, Ruminy P, Laé M. Detection of sarcoma fusions by a next-generation sequencing-based ligation-dependent multiplex RT-PCR assay. Mod Pathol. 2022;35:649–63. 10.1038/s41379-021-00980-x. 35075283

[47] Li X, Anand M, Haimes J, Manoj N, Berlin A, Kudlow B, Nucci M, Ng T, Stewart C, Lee C. The application of next-generation sequencing-based molecular diagnostics in endometrial stromal sarcoma. Histopathology. 2016;69:551–9. 10.1111/his.12966. 26990025

[48] Linardic CM. PAX3-FOXO1 fusion gene in rhabdomyosarcoma. Cancer Lett. 2008;270(1):10–8. 10.1016/j.canlet.2008.03.035 . Epub 2008 May 23. PMID: 18457914; PMCID: PMC2575376. 18457914 PMC2575376

[49] Lopes-Brás R, López-Presa D, Esperança-Martins M, Melo-Alvim C, Gallego L, Costa L, Fernandes I. Genomic profiling of sarcomas: A promising weapon in the therapeutic arsenal. Int J Mol Sci. 2022;23. 10.3390/ijms232214227. PMC9693140 36430703

[50] Mercatali L, Vanni S, Miserocchi G, Liverani C, Spadazzi C, Cocchi C, Calabrese C, Gurrieri L, Fausti V, Riva N, Genovese D, Lucarelli E, Focarete ML, Ibrahim T, Calabrò L, De Vita A. The emerging role of cancer nanotechnology in the panorama of sarcoma. Front Bioeng Biotechnol. 2022;10:953555. 10.3389/fbioe.2022.953555 . PMID: 36324885; PMCID: PMC9618700. 36324885 PMC9618700

[51] Miettinen M, Abdullaev Z, Turakulov R, Quezado M, Luiña Contreras A, Curcio CA, Rys J, Chlopek M, Lasota J, Aldape KD. Assessment of the utility of the sarcoma DNA methylation classifier in surgical pathology. Am J Surg Pathol. 2024;48(1):112–22. 10.1097/PAS.0000000000002138. 37921028 PMC10842611

[52] Miele E, De Vito R, Ciolfi A, Pedace L, Russo I, De Pasquale MD, Di Giannatale A, Crocoli A, Angelis B, Tartaglia M, Alaggio R, Milano GM. DNA methylation profiling for diagnosing undifferentiated sarcoma with Capicua transcriptional receptor (CIC) alterations. Int J Mol Sci. 2020;21(5):1818. 10.3390/ijms21051818. 32155762 PMC7084764

[53] Mosele MF, Westphalen CB, Stenzinger A, et al. Recommendations for the use of next-generation sequencing (NGS) for patients with advanced cancer in 2024: A report from the ESMO Precision Medicine Working Group. Ann Oncol. 2024. 10.1016/j.annonc.2024.04.005. 38834388

[54] Nacev B, Sánchez-Vega F, Smith S, Antonescu C, Rosenbaum E, Shi H, Tang C, Socci N, Rana S, Gularte-Mérida R, Zehir A, Gounder M, Bowler T, Luthra A, Jadeja B, Okada A, Strong J, Stoller J, Chan J, Tap W. Clinical sequencing of soft tissue and bone sarcomas delineates diverse genomic landscapes and potential therapeutic targets. Nat Commun. 2021;13:30453. 10.1038/s41467-022-30453-x. PMC9200818 35705560

[55] Öfverholm I, Wallander K, Haglund C, Chellappa V, Wejde J, Gellerbring A, Wirta V, Renevey A, Caceres E, Tsagkozis P, Mayrhofer M, Papakonstantinou A, Linder-Stragliotto C, Bränström R, Larsson O, Lindberg J, Lin Y, Haglund de Flon F. Comprehensive genomic profiling alters clinical diagnoses in a significant fraction of tumors suspicious of sarcoma. Clin Cancer Res. 2024;30(12):2647–58. 10.1158/1078-0432.CCR-24-0384. 38573684

[56] Patton A, Dermawan JK. Current updates in sarcoma biomarker discovery: emphasis on next-generation sequencing-based methods. Pathology. 2024;56(2):274–82. 10.1016/j.pathol.2023.10.015. 38185613

[57] Pestana C, Groisberg R, Roszik J, Subbiah V. Precision oncology in sarcomas: Divide and conquer. JCO Precision Oncol. 2019;3. 10.1200/po.18.00247. PMC7446356 32914012

[58] Racanelli D, Brenca M, Baldazzi D, Goeman F, Casini B, De Angelis B, Guercio M, Milano G, Tamborini E, Busico A, Dagrada G, Garofalo C, Caruso C, Brunello A, Pignochino Y, Berrino E, Grignani G, Scotlandi K, Parra A, Maestro R. Next-generation sequencing approaches for the identification of pathognomonic fusion transcripts in sarcomas: The experience of the Italian ACC Sarcoma Working Group. Front Oncol. 2020;10. 10.3389/fonc.2020.00489. PMC7175964 32351889

[59] Rekhi B, Upadhyay P, Ramteke MP, Dutt A. MYOD1 (L122R) mutations are associated with spindle cell and sclerosing rhabdomyosarcomas with aggressive clinical outcomes. Mod Pathol. 2016;29(12):1532–40. 10.1038/modpathol.2016.144. 27562493 PMC5133269

[60] Remiszewski P, Falkowski S, Szumera-Ciećkiewicz A, Spałek MJ, Rutkowski P, Czarnecka AM. Detection of gene fusions in soft tissue sarcoma using next-generation sequencing. Genes. 2026;17(5):514. 10.3390/genes17050514. 42194972 PMC13206013

[61] Remiszewski P, Siedlecki E, Kamińska M, et al. Choosing the right animal model for sarcoma research. Cell Mol Life Sci. 2026b;83., Article 1. 10.1007/s00018-025-06013-z. PMC12852540 41513885

[62] Remiszewski P, Tysarowski A, Szumera-Ciećkiewicz A, et al. Clinicopathological and genomic profiling in undifferentiated pleomorphic sarcoma. J Appl Genet. 2025. 10.1007/s13353-025-01036-5. 41413689

[63] Robinson DR, Wu YM, Kalyana-Sundaram S, et al. Identification of recurrent *NAB2*-*STAT6* gene fusions in solitary fibrous tumor by integrative sequencing. Nat Genet. 2013;45(2):180–5. 10.1038/ng.2509. 23313952 PMC3654808

[64] Schlieben LD, Carta MG, Moskalev EA, Stöhr R, Metzler M, Besendörfer M, Meidenbauer N, Semrau S, Janka R, Grützmann R, Wiemann S, Hartmann A, Agaimy A, Haller F, Ferrazzi F. Machine learning-supported diagnosis of small blue round cell sarcomas using targeted RNA sequencing. J Mol Diagn. 2024;26(5):387–98. 10.1016/j.jmoldx.2024.02.002. 38395409 PMC12178384

[65] Schöpf J, Uhrig S, Heilig CE, et al. Multi-omic and functional analysis for classification and treatment of sarcomas with *FUS*-TFCP2 or *EWSR1*-TFCP2 fusions. Nat Commun. 2024;15:51. 10.1038/s41467-023-44360-2. 38168093 PMC10761971

[66] van de Haar J, Roepman P, André F, et al. ESMO Recommendations on clinical reporting of genomic test results for solid cancers. Ann Oncol. 2024;35(11):954–67. 10.1016/j.annonc.2024.06.018. 39112111

[67] van IJzendoorn DGP, Szuhai K, Briaire-de Bruijn IH, Kostine M, Kuijjer ML, Bovée JVMG. Machine learning analysis of gene expression data reveals novel diagnostic and prognostic biomarkers and identifies therapeutic targets for soft tissue sarcomas. PLoS Comput Biol. 2019;15(2):e1006826. 10.1371/journal.pcbi.1006826. 30785874 PMC6398862

[68] Vanni S, Fausti V, Fonzi E, et al. Unveiling the Genomic Basis of Chemosensitivity in Sarcomas of the Extremities: An Integrated Approach for an Unmet Clinical Need. Int J Mol Sci. 2023;24(8):6926. 10.3390/ijms24086926. Published 2023 Apr 8. 37108089 PMC10138892

[69] Wallander K, et al. Sarcoma care in the era of precision medicine. J Intern Med. 2023. 10.1111/joim.13717. 37643281

[70] Wang D, Tu J, Liu J, Piao Y, Zhao Y, Xiong Y, Wang J, Zheng X, Liu B. Integrated multi-omics analysis and machine learning identify G protein-coupled receptor-related signatures for diagnosis and clinical benefits in soft tissue sarcoma. Front Immunol. 2025;16:1561227. 10.3389/fimmu.2025.1561227. 40761790 PMC12318986

[71] Weiss M, Blank A, Gitelis S, Fidler M, Batus M. Clinical benefit of next generation sequencing in soft tissue and bone sarcoma: Rush University Medical Center’s experience. J Clin Oncol. 2019. 10.1200/jco.2019.37.15_suppl.e22552.31800340

[72] WHO Classification of Tumours. In. Soft tissue and Bone. 5th ed. Lyon: IARC. 2020.

[73] Xerxa E, Koch S, Vanni S, De Vita A, Bajorath J. Drug sensitivity prediction across cancer cell lines with a focus on sarcoma. Artificial Intelligence in the Life Sciences. 2026;9:100157. 10.1016/j.ailsci.2026.100157.

[74] Zhao G, Xie L, Guo W, Xi Y, Cui Y, Yu S, Wan Q, Chen D, Pan X, Dong X, Wang K, Wang J. The impact of next generation sequencing on sarcoma diagnosis. J Clin Oncol. 2020. 10.1200/jco.2020.38.15_suppl.e23528.33119477

[75] Zhou C, Chen X, Huang Y, Zhang Q, Zhu S, Fu W. Nanomaterial Technology and Soft Tissue Sarcomas. Front Oncol. 2022;12:921983. 10.3389/fonc.2022.921983 . PMID: 35814363; PMCID: PMC9257037. 35814363 PMC9257037

[76] Zhu G, Benayed R, Ho C, Mullaney K, Sukhadia P, Rios K, Berry R, Rubin B, Nafa K, Wang L, Klimstra D, Ladanyi M, Hameed M. Diagnosis of known sarcoma fusions and novel fusion partners by targeted RNA sequencing with identification of a recurrent ACTB-FOSB fusion in pseudomyogenic hemangioendothelioma. Mod Pathol. 2018;32:609–20. 10.1038/s41379-018-0175-7. 30459475 PMC6486453

[77] Zhu Z, Zhou Q, Sun Y, Lai F, Wang Z, Hao Z, Li G. MethMarkerDB: a comprehensive cancer DNA methylation biomarker database. Nucleic Acids Res. 2024;52(D1):D1380–92. 10.1093/nar/gkad923. 37889076 PMC10767949

[78] Zugman M, Fernandes M, De Lima L, Teixeira M, Xavier T, Dias I, Pelegrino U, Côrtes K, Silva L, Silva D, Beal E, Uson J, Campregher P, Moura P, Filippi F, Jesus-Garcia R, R., Pestana R. Molecular profile and actionability of sarcomas with next-generation sequencing: Insights from a single institution in Brazil. ESMO Open. 2024. 10.1016/j.esmoop.2024.102522.

