# Supplementary material for: Next-Generation Sequencing in Translational Sarcoma Research: Biological Insights and Emerging Directions
Source: Curr Oncol Rep. 2026 Sep 17;28(1):96. doi: 10.1007/s11912-026-01828-w (PMC13585967; doi:10.1007/s11912-026-01828-w)
Supplement: Supplementary file 1 [file 11912_2026_1828_MOESM1_ESM.odt]

Supplementary Table 1: NGS relevance in sarcoma research

Key points
Supporting evidence
References
NGS enables comprehensive mutation and variant discovery in sarcoma
Multiple large studies show NGS detects a wide range of mutations and structural variants in sarcoma
Cote et al., 2018; Gounder et al., 2022; Bajpai et al., 2025; Nacev et al., 2021; Dorand et al., 2023
NGS is highly effective for fusion gene identification and discovery
RNA-based and anchored multiplex NGS panels detect known and novel fusions with high sensitivity
Lam et al., 2018; Gupta et al., 2025; Aggarwal et al., 2025; Racanelli et al., 2020; Lanic et al., 2022; Zhu et al., 2018; Hu et al., 2022
NGS facilitates the study of tumor heterogeneity and clonal evolution
Single-cell and longitudinal NGS studies reveal intra- and inter-tumor heterogeneity and evolution
Eisenhardt et al., 2022; Bajpai et al., 2025; Grachev et al., 2024; Grachev et al., 2025; Comeau et al., 2024
NGS supports biomarker and target discovery in sarcoma research
NGS-based profiling identifies actionable mutations and molecular signatures for research and drug development
Hensley et al., 2020; Dufresne et al., 2018; Eisenhardt et al., 2022; Bajpai et al., 2025; Lopes-Brás et al., 2022; Dorand et al., 2023; Zugman et al., 2024
NGS is essential for developing and validating new molecular assays
NGS platforms are used to create and validate sensitive, multiplexed assays for research applications
Lam et al., 2018; Gupta et al., 2025; Jeyaraman et al., 2025; Lanic et al., 2022; Cheng et al., 2021; Hu et al., 2022
Technical and interpretive challenges remain in NGS-based research
Issues include RNA degradation, data complexity, and need for standardized pipelines
Patton & Dermawan, 2024; Jeyaraman et al., 2025; Itkin et al., 2024; Pestana et al., 2019
