# Supplementary material for: Next-Generation Sequencing in Translational Sarcoma Research: Biological Insights and Emerging Directions
Source: Curr Oncol Rep. 2026 Sep 17;28(1):96. doi: 10.1007/s11912-026-01828-w (PMC13585967; doi:10.1007/s11912-026-01828-w)
Supplement: Supplementary file 2 [file 11912_2026_1828_MOESM2_ESM.odt]

Supplementary Table 1: NGS relevance in sarcoma research

Key points
Supporting evidence
References
NGS enables comprehensive mutation and variant discovery in sarcoma
Multiple large studies show NGS detects a wide range of mutations and structural variants in sarcoma
Cote et al., 2018 [16]; Gounder et al., 2022[30] ; Bajpai et al., 2025 [4]; Nacev et al., 2021[54]; Dorand et al., 2023 [22 ]
NGS is highly effective for fusion gene identification and discovery
RNA-based and anchored multiplex NGS panels detect known and novel fusions with high sensitivity
Lam et al., 2018 [45]; Gupta et al., 2025 [32]; Aggarwal et al., 2025 [1]; Racanelli et al., 2020 [58]; Lanic et al., 2022 [46]; Zhu et al., 2018 [75]; Hu et al., 2022 [37]
NGS facilitates the study of tumor heterogeneity and clonal evolution
Single-cell and longitudinal NGS studies reveal intra- and inter-tumor heterogeneity and evolution
Eisenhardt et al., 2022 [24]; Bajpai et al., 2025 [4]; Grachev et al., 2024 [29]; Grachev et al., 2025 [28]; Comeau et al., 2024 [14]
NGS supports biomarker and target discovery in sarcoma research
NGS-based profiling identifies actionable mutations and molecular signatures for research and drug development
Hensley et al., 2020 [35]; Dufresne et al., 2018 [23]; Eisenhardt et al., 2022 [24]; Bajpai et al., 2025 [4]; Lopes-Brás et al., 2022 [49]; Dorand et al., 2023 [22]; Zugman et al., 2024 [77]
NGS is essential for developing and validating new molecular assays
NGS platforms are used to create and validate sensitive, multiplexed assays for research applications
Lam et al., 2018 [45]; Gupta et al., 2025 [32]; Jeyaraman et al., 2025 [40]; Lanic et al., 2022 [46]; Cheng et al., 2021 [13]; Hu et al., 2022 [37]
Technical and interpretive challenges remain in NGS-based research
Issues include RNA degradation, data complexity, and need for standardized pipelines
Patton & Dermawan, 2024 [56]; Jeyaraman et al., 2025 [40]; Itkin et al., 2024 [39]; Pestana et al., 2019 [57]
